# Supplementary material for: Therapeutic Decision Making in Prevascular Mediastinal Tumors Using CT Radiomics and Clinical Features: Upfront Surgery or Pretreatment Needle Biopsy?
Source: Cancers (Basel). 2024 Feb 13;16(4):773. doi: 10.3390/cancers16040773 (PMC10886806; doi:10.3390/cancers16040773)
Supplement: Supplementary file 1 [file cancers-16-00773-s001.zip › cancers-2725795-supplementary.pdf]

Supplementary Table S1. The centrality and dispersion for age, LDH, AFP and HCG.

		Age	LDH	AFP	HCG
Category 1.	N	182	31	45	31
	Mean	60.02	210.10	2.46833	.40555
	Std. Deviation	15.629	91.214	1.299046	.905478
	Grouped Median	60.87	189.00	2.29000	.17167
	Kurtosis	.292	4.856	1.140	6.957
	Skewness	-.463	1.920	.766	2.679
	Range	87	423	6.470	3.700
Category 2.	N	193	116	82	68
	Mean	52.70	392.15	1128.34102	2994.00781
	Std. Deviation	20.945	376.722	6176.807435	21326.820646
	Grouped Median	54.50	250.50	2.10500	.12727
	Kurtosis	-1.054	6.735	53.231	65.321
	Skewness	.017	2.537	7.009	8.025
	Range	83	1979	50610.000	174608.000
Total	N	375	147	127	99
	Mean	56.25	353.76	729.40976	2056.61720
	Std. Deviation	18.890	345.034	4981.887025	17689.109293
	Grouped Median	59.14	238.00	2.21667	.14103
	Kurtosis	-.649	9.078	83.142	95.156
	Skewness	-.273	2.884	8.752	9.685
	Range	92	1979	50610.000	174608.000

Supplementary Table S2. List of clinical and radiomic features extracted by the third LASSO logistic regression analysis (Selection_3).

**Clinical feature**
LDH
**Radiomic features**
original_shape_Flatness
original_shape_Maximum2DDiameterSlice
original_shape_Sphericity
original_firstorder_10Percentile original_firstorder_Mean
original_gldm_DependenceNonUniformityNormalized
wavelet.LLH_firstorder_Energy
wavelet.LLH_firstorder_TotalEnergy
wavelet.LLH_glcm_ClusterProminence
wavelet.LLH_glcm_Idmn
wavelet.LLH_glszm_LowGrayLevelZoneEmphasis
wavelet.LLH_glszm_SmallAreaLowGrayLevelEmphasis
wavelet.LHH_glcm_MCC
wavelet.LHH_glszm_LowGrayLevelZoneEmphasis
wavelet.LHH_glszm_SizeZoneNonUniformityNormalized
wavelet.LHH_glszm_ZoneEntropy
wavelet.HLL_glcm_ClusterProminence
wavelet.HHL_glcm_Correlation
wavelet.HHL_glcm_InverseVariance
wavelet.HHL_glszm_LowGrayLevelZoneEmphasis
wavelet.LLL_glcm_InverseVariance
wavelet.LLL_glszm_SmallAreaEmphasis

Abbreviations: LDH: lactate dehydrogenase
